# Tailoring First Aid Courses to Older Adults Participants

**DOI:** 10.1177/10901981211026531

**Published:** 2021-08-05

**Authors:** Eva Dolenc, Marko Kolšek, Damjan Slabe, Ivan Eržen

**Affiliations:** 1 University of Ljubljana, Ljubljana, Slovenia

**Keywords:** aging and older adults, community health promotion, health education

## Abstract

Relevant organizations emphasize the importance of first aid (FA) for older adults due to the increased risk of injuries and sudden illnesses in old age. Even though FA training guidelines have been developed, no program for an FA course adapted for the older adults has been formally adopted in Europe. This study’s objective is to identify older adults’ needs, beliefs, desires, advantages, and possible limitations in connection with FA. This qualitative study used semistructured interviews with 22 laypersons and retired health professionals older than 60 years old. The qualitative content analysis indicated that the major themes elicited by the older adults are motivation to participate in the FA training, older adults’ specific features as a resource or obstacle for participating in FA training, general suggestions, and content suggestions for FA training. Older adults are very differently motivated to participate in FA training due to the heterogeneity of their psychophysical abilities. They need and want to obtain additional knowledge from the field of FA and health protection for which any psychophysical limitations are not as relevant as when learning cardiopulmonary resuscitation. They want to learn how to recognize emergency situations and more about calling emergency services with the use of modern technology. In addition to cardiopulmonary resuscitation without rescue breaths, they also want to learn about topics related to the treatment of injuries. Those who had practiced FA in their work–life think that they can be a good source to transfer their knowledge to persons from their generation. While planning an FA training course, it has to be taken into consideration that older adults want a short course, adjusted to their varied psychophysical abilities. Due to the wide array of contents they want to learn, it would be reasonable to prepare a selection of different programs for short training courses.

The health of older adults has a decisive influence on the aging trend (World Health Organization, 2015). In general, older adults, women, children and disabled people are vulnerable groups of the population, resulting in a lower capacity of adaptation and survival in emergencies and disasters (American Red Cross, 2020b; Sheikhbardsiri et al., 2017; World Health Organization, 2015). The increasing risk of injuries and sudden illnesses (Marengoni et al., 2011; World Health Organization, 2015) are some of the reasons that relevant organizations (American Red Cross, 2020a; World Health Organization, 2015; International Federation of Red Cross and Red Crescent Societies, 2016) emphasize the importance of first aid (FA), especially cardiopulmonary resuscitation (CPR), also for older adults. Most cardiac arrest victims are in their late 60s; 85% of them collapse at home, and half are witnessed by a family member or friend who is usually older than the age of 55 years (Vaillancourt et al., 2004). The number of out-of-hospital cardiac arrests in 2020 increased; this is significantly correlated to the COVID-19 pandemic and is coupled with a reduction in short-term outcomes (Baldi et al., 2020). With the poorer access to emergency medical services (Katayama et al., 2020) and due to changes in health care–seeking behavior (Hughes et al., 2020) during the COVID-19 pandemic, the importance of FA knowledge of the older people increases even more.

The first evidence-based guidelines for FA were developed by the American Heart Association in Collaboration with the International Liaison Committee on Resuscitation (2000). In 2015, the European Resuscitation Council (2015) included a chapter on FA in the *European Resuscitation Council Guidelines for Resuscitation*. In addition to CPR, there is also content about FA for medical and trauma emergencies, which are the skills that older adults must also acquire. The European Resuscitation Council concluded that FA education programs, public health campaigns, and formal FA training are recommended for improving the prevention, recognition, and management of injury and illness. The International Federation of Red Cross and Red Crescent Societies highlighted the importance of FA knowledge for older people by dedicating the World First Aid Day in 2015 to older populations (International Federation of Red Cross and Red Crescent Societies, 2015). FA training is also an example of a multidimensional intervention that contains cognitive training and physical activity, which can be an effective way (among others) of reducing frailty (Veninšek & Gabrovec, 2018).

In contrast, Brinkrolf et al. (2017) determined that people older than the age of 65 years have less FA knowledge than younger people do, especially about CPR. There is also a strongly decreasing level of knowledge and willingness to use automatic external defibrillators (AEDs) with increasing age (Krammel et al., 2018). After examining practical knowledge, Richman et al. (2007) concluded that many members of their sample failed to adequately perform some key steps in the simulated cardiac arrest scenario. Consequently, older adults have lower confidence in their ability to apply resuscitation than younger people do (Brinkrolf et al., 2017). In a real situation of cardiac arrest, Park et al. (2020) determined that people older than the age of 60 years perform CPR at a lower quality. Takei et al. (2014) confirmed that older eyewitnesses are less likely to perform CPR in reality. One possible reason is that a smaller proportion of older adults received FA training (Dobbie et al., 2018), which is a proven incentive to provide FA (Bakke et al., 2015; Tanigawa et al., 2011). They also have a poorer psychophysical condition (Panday et al., 2019). Some people worry about their physical capabilities to learn FA techniques such as CPR, and it was a particular concern for older respondents (Potts & Lynch, 2006). Poor health is also one of the reasons why older adults had not sought CPR training. Health conditions mentioned included arthritis, joint replacements, lung conditions, or the perceived general inability to provide the effort necessary to do CPR (Vaillancourt et al., 2013). It should be considered that multimorbidity is present in more than 60% of older adults (Barnett et al., 2012). The number of people with functional decline or disability in old age is increasing (Robnett et al., 2020). Frailty, which is significantly associated with morbidity and mortality, is another specific problem among older adults (Kojima et al., 2018; Veninšek & Gabrovec, 2018). Nevertheless, older adults also want to learn or refresh their FA knowledge (Dolenc et al., 2018). There have already been some successful FA training sessions for older adults in various countries (Burton et al., 2017; Neset et al., 2010). Most of those that provided FA courses focused on CPR and falls. However, there are many more health complications in old age and the consequent need for FA measures. In 2012, Monette (2012) warned of an urgent need for a systematic approach, and FA courses for older adults are advised. Public health interventions targeting this population would represent the best strategy, emphasizes Vaillancourt et al. (2013).

It is important to identify unique social, personal, and environmental factors that inhibit or promote a layperson’s behavior that protects or enhances health (Grimes et al., 2020). Heard et al. (2020), in their scoping review, identified the following barriers to the uptake of FA training among laypersons of all ages: a failure to realize that FA skills can save lives, a lack of available or convenient FA classes, cost, an aversion to or fear of taking responsibility, and a fear of infection. In contrast, a belief that FA skills can save a life, especially life of a family member, increases the uptake. Previously trained respondents were more likely to express interest in future training, as were people who had witnessed a cardiac arrest (Schmid et al., 2016). Vaillancourt et al. (2013) identified key facilitators and barriers for CPR training and performance in a purposive sample of laypersons aged 55 years and older. Facilitators of CPR training included classes in a convenient location, more advertisements, and having a spouse. Barriers to taking CPR training included perception of physical limitations, time commitment, and cost. The results of a Canada-wide study indicate that, in order to convince more people older than the age of 55 years to complete a CPR class, public health interventions need to target their attitudinal beliefs. People who are most likely to adopt these behaviors already know that CPR can save someone’s life; that satisfaction can be gained from mastering this skill; that CPR should be initiated without delay, even before emergency medical service arrival; and that they are unlikely to get in trouble for attempting to help someone (Vaillancourt et al., 2013). This phenomenon is addressed by the theory of planned behavior (TPB; Ajzen, 1991), which assumes that the strength of an individual’s intention to engage in a behavior (in the case of the topic of our research, participation in FA training for older adults) and the degree of control they feel they have over that behavior are the proximal determinants of engaging in that behavior. Intention is influenced by attitudes/behavioral beliefs, subjective normative beliefs, and control beliefs. Measuring an individual’s intention to engage in a particular behavior has been shown to correlate well with performing the behavior (Ajzen, 1991).

Regarding FA, Krammel et al. (2018) suggest that specifically tailored programs to increase the awareness and willingness in the older adults community need to be considered for future educational interventions. Unfortunately, no such program for an FA course, adapted for the older adults, through the analysis of European FA courses, exists (Dolenc et al., 2018). Furthermore, we do not know what the beliefs, needs, and desires of older adults are as far as FA is concerned. This study explores (Slovenian) older adults’ opinions about an adapted FA course for them. The purpose is to identify how the program should be tailored for older adults to acquire the FA skills they need. The findings presented in this article are a continuation of efforts to develop a formally adopted FA course adapted for older adults.

## Method

### Study Design

This qualitative study used semistructured interviews to explore older adults’ opinions about FA. We had followed the 32-item consolidated criteria checklist for reporting qualitative research (Tong et al., 2007). The study’s objective is to identify the older adults’ beliefs, needs, desires, advantages, and possible limitations in connection with FA. The goal of the research was to use the findings to prepare a program for an FA training course adapted for older adults.

### Selection of Study Participants

Through purposive sampling, participants were recruited from the population of Slovenian older adults. The following sampling criteria were applied: participants from different age groups (60–69, 70–79, 80–89, and 90–100 years old); half of the sample consisted of (retired) health care professionals (doctors, nurses) or in regular contact with FA (mountain rescuers, responsible for FA field at the Red Cross). The other half of the sample consisted of laypersons defined as individuals from different nonmedical professions and not in contact with FA (teachers, factory worker, flight attendants). Contacts of potential participants were provided by coauthors who did not conduct the interview. Participants were approached face-to-face or by telephone; two refused to participate due to illness. All interviews were conducted face to face. No relationship with the interviewer was established prior to the study commencement. We introduced the researchers to them and explained the purpose of the research. We also checked that all of the participants were willing to participate in our study; all of them agreed; they signed a declaration of voluntary participation. The purposive sample size was determined by theoretical saturation, which is the point in the data collection process when new data no longer offer additional insights for the research question (Glaser & Strauss, 1999). The final number of interviews conducted was 22 (Table 1).

**Table 1.** Demographic Data of the Interviewees.

Demographic data		*n*
Gender		
Male		8
Female		14
Age-group (years); mean age is 76 years		
60–69		8
70–79		4
80–89		8
90–100		2
Profession/activity; most (16) of the participants are retired		
Health care professionals or FA activity (one interviewee has several professions or is involved in several FA activities)	Nurse (two district nurses: one was employed in a nursing home and one in hospital)	4
	Doctor (three family physicians: one of them is also professor of the history of medicine)	3
	Licensed FA lecturer	3
	Pharmacist	2
	Psychologist	2
	Mountain rescuer	1
	Director of a sports medicine hospital	1
	Responsible for FA at the Red Cross	1
Layperson	Factory worker	8
	Teacher	3
	Flight attendant	1
	President of the mountaineering association	1
Education		
Primary school		8
College: University—master or PhD		14
Place of residence		
City		8
Countryside		14

*Note.* FA = first aid.

### Qualitative Method

In line with the research goals, we decided to conduct interviews to explore older adult’s opinions. A semistructured questionnaire was prepared and developed through a literature review (first and last author). It was additionally redesigned after pilot interviews (first and third author). The questionnaire (see Supplemental Appendix A) consisted of 15 questions. It was divided into sections: the attitude toward FA and health (introductory questions), FA experience, and FA training. The interviews were conducted by the first author, a doctoral student of Public Health at the Faculty of Medicine, University of Ljubljana, and a teaching assistant for FA. She had individual training on qualitative methodology, which was led by the second and third authors. The second author is a teacher in qualitative methods for doctoral students. The interviews were held face-to-face between February and August 2020. They took place at the interviewee’s home, without the presence of other persons. The interviews were recorded digitally (audio), transcribed verbatim, and anonymized. Some field notes were made during and after the interview and were included in the final analysis. Transcripts were not returned to participants. We did not make repeat interviews.

### Outcomes and Analysis

The first, second, and third authors conducted a qualitative content analysis. The interview transcripts were read, qualitatively coded, reviewed, and labelled, using inductive content analysis (Elo & Kyngäs, 2008). Each interview was analyzed by the authors independently and by generating codes for each statement reflecting the older adult interviewees’ opinions. After individual analysis, the authors reached a consensus on categories and themes. Themes and categories were defined in an inductive process after establishing codes to condense observations from the data. Themes and categories had not been predetermined. To secure the anonymity, interviewees’ names were changed, but they did not yet provide feedback on the findings.

## Results

The interviews lasted from 6 to 64 minutes. We assume that interviewees’ responses varied in time because of the heterogeneity in their psychophysical capabilities, health status, experiences, and pre-education. Based on content analysis, we grouped the codes into categories and four themes. We determined the following themes: motivation to participate in the FA training, older adult’s specifics as a resource or obstacle for participating in FA training, general suggestions for older adult’s FA training, and content suggestions for such training.

### Motivation to Participate in the FA Training

Table 2 shows that the interviewed older adults are differently motivated to participate in the FA training. Angela (83 years) says, “Oh, come on, I’m an old woman. No, that’s for young people. We can’t do it.” In contrast, Jose (81 years) says, “Well, I think there shouldn’t be an age limit. I’m 81, and I feel if I were encouraged to do that, why not, it’s useful.” They also state some motivational factors, including social and legal aspects. Ton (82 years), as a retired doctor, suggests,

In my experience, there would be a revolt if we adjusted the Road Traffic Safety Act and made it mandatory for people to take a FA course again when they renew their driver’s license. If the politicians passed that law, it could be a short course, perhaps just a chat during the physical examination, something like, if you come across an accident, do this and this. We can do enough if we remind people which telephone number to call.

**Table 2.** Categorized Interviewees’ Answers Regarding the Older Adults’ Motivation to Participate in an FA Course.

Category	Subcategory	Codes (examples)
In favor of training	Importance of knowledge	Everyone should have some knowledge.
	Expressed interest	We are interested, a course should be offered.
	Expressed rationality and a positive attitude	Lifelong learning program necessary; it makes you feel important because you can take care of yourself and others; everyone older than 60 years should know the basics.
	Social encouragement	We live with grandchildren and babysit them, so we wish to know how to react.
	Legal obligation	Yes. Revise the Road Traffic Safety Act and set a rule that the FA knowledge should be reviewed when renewing the driver’s license.
	Effect	It slows down the effects of dementia. Not to make it too demanding, because there will be no effect.
Conditionally in favor of training	Voluntary participation	If there is interest, then yes for those who wish it.
	Yes, until 80 years, not later	Yes, until 80 years of age. Later it would be difficult, depending on age.
	Necessary adjustments	Older adults will be happy to participate if the program is suitably adjusted
Not in favor of training	Not ready to participate	I don’t need it, I wouldn’t attend; I don’t think it’s important; I should but I’m not suitable for that.
	Age as a reason	What’s the point in old age.
	Assigning responsibility to others	It’s for the young; if people are old, relatives should attend.
	Not organized	Conditions are not good.

*Note.* FA = first aid.

Older adults who are retired health care workers and who had practiced FA in their professions, can be a good source to transfer their experience and knowledge to persons from their generation. Sarko (64 years) says, “This is my final year of employment—then comes retirement. I’ve been working with FA teams from schools, Red Cross teams, Civil protection teams, etc. Now I can work with older adults—we will fit together better.”

### Older Adults’ Specific Features as a Resource or Obstacle for Participating in FA Training

Heterogeneity in the psychophysical capabilities of the older adults influences their capability of administering FA and their motivation to participate in FA courses. Ton (82 years) says, “I think I could still perform CPR.” In contrast, Ani (96 years) says, “You know, I had a stroke about a month ago. I forgot so many things; I hardly know where I live. I’m quite crazy, but not completely.” Ton (82 years), as a retired doctor, stresses, “In an old person it depends a lot on how his senses work.” Berna (68 years) speaks from personal experience:

I run a group called older adult for self-help, a group of older adults in the Organization of Disabled Persons, a group of volunteers older adults for older adults . . . They tell me how they treated injuries in their youth, and they want to know, how to react now. (Table 3)

**Table 3.** Categorized Interviewees’ Answers Regarding the Older Adults’ Psychophysical and Other Specifics.

Category	Subcategory	Codes (examples)
Sufficient psychophysical capabilities	Strength	I have the strength; I could perform CPR.
	Activity	Some are very active.
	Psychical condition	You are of sound body and mind.
	Age	There are exceptions, but one can still do it at 75 or 80 years.
Insufficient psychophysical capabilities	Inability—general	You cannot do it.
	Physical limitations	My legs hurt, I weigh 42 kg.
	Psychical limitations	Some people can concentrate, others not.
	Senses	A lot depends on the functionality of the senses; some are hard of hearing, vision impaired, etc.
	Forgetfulness	I am so forgetful that I don’t know where my home is.
	Age	People older than 80 years are less capable.
Resources	Relativity of age	80-Year-olds can be very teachable, and a 50-year-old can refuse to learn.
	Experience	They have experience.
	Prior knowledge	Some things are retained.
	Ability to use information–communication technology	They got used to computers.
	Interest	Some are interested.
	Prudence	They are more prudent.
Obstacles	Prejudice	The young have prior knowledge, while the older adults don’t.
	Fear	You get scared, you panic.
	Accessories	Crutches and walking frame.
	Sickness	Very demented.

*Note.* FA = first aid; CPR = cardiopulmonary resuscitation.

### General Suggestions for Older Adults’ FA Training

The interviewees gave many suggestions on preparing FA courses for older adults (Table 4). Meta (80 years) worked on preparing FA training programs:

Not an extensive program, just a few measures. We would have to prepare a few short notes for them, stating only a few life-saving measures, so that they can read them, perhaps a booklet. We have to start somewhere. Take the 10-hour Red Cross program for drivers. Adapt it. No need to reinvent the wheel.

Ani (70 years), who used to work as a nurse in a retirement home, warns about the need to be moderate:

Up to a certain age, you give to the society, after that you receive. We mustn’t bombard the older adults with knowledge that they no longer need. The young people need lots of knowledge. The older adults need to learn to recognize some signs and remember to call 112.

**Table 4.** Categorized Interviewees’ Answers Regarding the General Suggestions for Older Adults’ FA Training.

Category	Subcategory	Codes (examples)
Course suggestions Equipment	Length	Short; not a lot; not more than 2 hours
	Invitations	First written invitations; invitations are distributed in apartment buildings
	Contractors	Depends who will hold courses–through various associations
	Teaching aids	A short booklet, given in advance.
	Technology	Including technology; there are a lot of defibrillators around
	Psychological aspects of learning	Reducing fear, empowering, and increasing the older adults’ self-image
	Learning philosophy	Children should have learned FA
Methodology suggestions	Learning methods	To check what they already know, warn of mistakes, add what is new; novelties; short talks; to show on the mannequin; discussion; teaching by examples, if, e.g., a grandchild falls; smaller groups; a lot of practical examples; no videos; in a group with a mentor; lectures
	Permanent learning	Permanent—building on previous knowledge
	Contents concept	FA basics; not exaggerate; informational; basic; a few measures; adaptation of the 10-hour course for drivers
	Adaptations	To adapt the teaching methods
Conceptual suggestions	Alternative teaching methods	It could be after the main news; formally adopted customized program
	Theories	Related to general systems theory (Bertalanffy’s general systems theory)

*Note.* FA = first aid.

### Content Suggestions for Older Adults’ FA Training

The interviewees’ opinion is that the FA training course needs to include several topics (Table 5). Mila (67 years) said, “Each topic can be brought closer to the older adult. Even if only three or five attend. It doesn’t matter.” The interviewees assert the importance of teaching them how to use modern technology. Ton (82 years) said,

Just, simply, take a mobile phone, or have a button on the phone, press it, they will pick up, and you say there has been a horrible accident and they will know where. They can figure it out; so don’t disconnect.

JOSI (61 years) said,

Well, maybe the older adult should be taught how to use the defibrillators, which are everywhere. They get donated, sometimes it’s a bit competitive; but we should know where they are located in our area, where you work and live, you should know the location of the nearest defibrillator.

**Table 5.** Categorized Interviewees’ Answers Regarding the Content Suggestions of the FA Training.

Category	Subcategory	Codes (examples)
FA contents	Call 112	Tell them whom to call; call 112 immediately; tell them there’s been an accident; don’t hang up immediately, you have to go ask if anything is needed, tell others to call; what can a dispatcher do; how to call; how 112 works; a call to 112
	Use of automatic external defibrillator	Bring a defibrillator and show how it works; tell people where defibrillators are
	CPR	Tell them the chance of survival has increased; check for breathing; mouth-to-mouth resuscitation; at least show the reanimation process; the older adults can attend CPR classes
	Identifying and monitoring life functions	Monitoring life functions
	Urgent conditions	Recognizing conditions; heart attack, stroke—signs
	Recovery position	Placing an unconscious person into the recovery position; proper recovery position
	Bandaging	Bandaging; use of triangular bandage; trauma dressing
	FA for bleeding	How to stop the bleeding; stopping the blood; how to stop the bleeding if you cut yourself
	Managing injuries	About injuries, wounds, and burns; how to cool down burns; recognizing wounds; cooling of burns
	Fractures	Broken arm, immobilization of smaller injuries on the arm
	Advising others, when the older adults cannot perform the measures	To help by advising
	Psychological FA	What they can do themselves; don’t forget about the psychological FA; they can provide a sense of safety
Contents about health	Therapeutic exercise	To show easy exercises—therapeutic exercises
	Taking care of health	To tell them something about health—how to take care of one’s health, nutrition, and sleep; raising awareness on nutrition and movement; talking about health; a healthy way of life; nutrition and movement; giving up bad habits and rest
Contents about illness	Medicines	Taking medication
Contents on preventive measures	Prevention	Preventing accidents and injuries
	Falls	Falls; how to react if one falls
Contents on technology	Use of mobile phone	Telephone, difficult to use; to have only one button on the phone to call
	Use of technology	The technology progresses; about smart cars; use of modern telecommunication devices when you call; bracelets if you fall
Unadvisable/unsuitable contents	No mouth-to-mouth resuscitation	Don’t talk about mouth-to-mouth resuscitation; it is secondary; no mouth to mouth
	No immobilization of legs	No immobilization; nothing about setting the bones

*Note.* FA = first aid; CPR = cardiopulmonary resuscitation.

## Discussion

The main finding of our study is that older adults are motivated to participate in FA training in diverse ways, due to the heterogeneity of their psychophysical abilities. Predominantly, due to reduced psychophysical abilities, illnesses, and other limitations, brought about by old age, many would choose not to participate in FA training. We have determined that health care workers have more positive statements in favor of FA training compared with non-health-related workers. Specifically, all 17 health care workers interviewed favored training and would also attend the training themselves, even if they are 80 years or older. Among the non-health care workers (laypersons) included in our research, only 5 out of 13 would attend the training, but they also believe that other older adults should be trained in FA if they are under the age of 80 years and psychophysically fit. Nevertheless, the majority of interviewees, especially those under 80 years of age, showed interest in participating in the FA training. Our research confirms the finding that older adults’ motivation to participate in an FA training course varies greatly. The results of other research studies show that older adults are less motivated to participate in an FA training course than younger people are (Dolenc et al., 2018). In contrast, many older adults do take part in training courses (Burton et al., 2017; Neset et al., 2010).

When considering options for increasing the uptake of FA training, it is important to recognize the barriers to training that are identified by the public and to develop ways to reduce them (Heard et al., 2020). According to TPB (Ajzen, 1991), we identified some factors that could influence the intention to participate in FA training for older adults. Interviewed older adults attach importance to FA, because of the deterioration in health conditions of their relatives or themselves, and especially because of chronic diseases. They believe that FA “can help me or my loved one.” In TPB, this is placed in predictive variable attitudes/behavioral beliefs. A related CPR-targeted study (Vaillancourt et al., 2013) came to the same conclusions. Regarding the subjective norms/social component of the TPB, several of our interviewed mentioned that they babysit their grandchildren, who have a greater possible risk of injuries and poisoning. Vaillancourt et al. (2013) stated that some participants were concerned about not knowing what to do if a grandchild were to drown or choke. Considering the control beliefs associated with FA training, psychophysical limitations notwithstanding, older adults already have rich practical experience in FA with injuries and sudden illnesses. Regarding the CPR course, Vaillancourt et al. also indicate the significance of location, promotion (motivational media advertisement), time, and costs. Heard et al. (2020) named the barriers to participation in FA training as logistical and economical. They mention that people report it is difficult to find a nearby FA training that fits into their schedules and their budgets. We find that this dictates a need for an adapted FA training for older adults that would consider their experiences, over which the adults also stress.

Older adults in our research emphasize that a shorter, basic course would be suitable for them. The guidelines for resuscitation (European Resuscitation Council, 2015; Greif et al., 2015) suggest that the CPR curriculum should be tailored to the target audience and kept as simple as possible. Course organizers have to plan their courses in a flexible way, allowing for a shorter duration for target groups with different backgrounds and more hands-on time for laypersons. Although there is some evidence that higher frequency (Woollard et al., 2006), short-burst training (Andresen et al., 2008) could potentially enhance basic life support training and reduce skill decay, more studies are needed to confirm this (European Resuscitation Council, 2015; Greif et al., 2015). As memory and the ability to learn on average decrease with age (Geda et al., 2011), the knowledge of FA should be renewed in the older population even more frequently. However, due to poorer psychophysical condition (Panday et al., 2019) and reduced ability to concentrate (Esteves et al., 2019), FA refresher courses should be shorter and focused. According to our interviewees, the course must include a lecture, discussion, and practical exercises. They stress that older adults need to be empowered by looking for resources (e.g., experience, previous knowledge) and by encouraging them to realize that they can still help their fellow man in some way, despite any limitations they may have. In one of our interviews, a retired health care worker offered an interesting idea. He believes that he himself could teach other older adults about FA. On this topic, the American Red Cross (2020a) published a manual titled *Disaster Preparedness for Seniors by Seniors*. A similar approach is practiced in Japan: community health workers, often called “health promotion volunteers,” are individuals who act as a natural helping resource in the community. Murayama et al. (2020) provide evidence that a community health care worker based natural helping approach is a possible solution to promote healthy aging in the community. A senior promoter can help peers overcome barriers. He can tell by his example that they are capable of some FA procedures. Almost every layperson is capable of dialing 112; therefore, they want to learn more about calling emergency medical services. Some time should be allocated for the technical aspects of making a call (use of a mobile phone with a call button, hands-free function). The older adult must recognize the situations for which they have to call 112 immediately (e.g., loss of consciousness, a stroke, acute coronary syndrome, severe bleeding, etc.). In addition to CPR using an AED, but without mouth-to-mouth resuscitation, they also want to learn about other FA topics, related to the treatment of injuries, especially stopping bleeding and treating wounds. Regarding fractures, the older adults want to focus on upper limbs, but not on immobilization of leg fractures. In addition to FA, they are also interested in several other health topics in old age, such as the prevention of falls and general medicine.

The majority of other existing research studies evaluating FA training courses for older adults or the physical abilities of older adults are focused on basic resuscitation procedures—that is, either on the knowledge or the ability to perform them. Comparable with our research, the other studies ascertain different levels of psychophysical abilities to perform some of the FA measures (CPR). Mannequin studies on 19 older adult laypersons (with a median age of 78 years) performing CPR have shown disappointing results, with very low CPR quality and poor skill retention (Dorph et al., 2003), which is also confirmed by some others (Park et al., 2020; Takei et al., 2014). In contrast, Neset et al. (2010) determined that laypersons aged 50 to 76 years are capable of performing 10 minutes of CPR with satisfactory quality. The reasons for the conflicting results can be found in the diverse psychophysical and other characteristics of older adults. The interviewees in our research told us that they do not want to learn mouth-to-mouth resuscitation. Liu et al. (2016) tested participants older than 55 years (mean age 70.8 years) without physical limitations and found out that CPR quality is better while performing only chest compressions. During the COVID-19 pandemic, resuscitation guidelines have been adapted regarding mouth-to-mouth resuscitation (Nolan et al., 2020). They also stress the use of a phone with a hands-free option to communicate with the emergency medical dispatch center during CPR (Nolan et al., 2020). Nebsbjerg et al. (2018) found inferior dispatcher-assisted CPR among older adults in a simulated setting but the potential for improvement after a training course in this age-group. As an addition to dispatcher-assisted CPR, Krikscionaitiene et al. (2016) added a four-hand chest compression (“Andrews maneuver”) as an efficient solution for improving the performance of older adult rescuers and helping them achieve the recommended chest compression depth.

To summarize, older adult laypersons are often in the frontline facing out-of-hospital cardiac arrest, while most training efforts are directed toward younger people (Nebsbjerg, 2018). The value of CPR training for older adults is great (Burton et al., 2017; Neset et al., 2010). The interviewees in our research believe that CPR knowledge is useful, and they are interested in learning CPR. However, it has to be emphasized that in addition to CPR, they need and want to obtain additional knowledge about FA and protection of health, for which any psychophysical limitations are not as relevant as when learning CPR. While planning an FA training course for the older adults, the authors believe that it has to be taken into consideration that the older adults want a short course adjusted to their varied psychophysical abilities. Due to the wide array of content that the older adults want to learn, it would be sensible to prepare a selection of different programs for short training courses: CPR with AED, FA measures for most common health complications in old age, and general health care. Every course should include instructions on how to make a call to emergency medical services. The older adults would agree to attend training in accordance with a program adjusted to their needs and abilities. Based on our findings, we designed a checklist of crucial items (see Supplemental Appendix B) that can help you when planning future intervention (FA course for older adults) or future research.

## Limitations and Recommendations for Future Research

The general limitation is related to the characteristics of qualitative research—for example, limited generalization of results due to sample size. Purposeful sampling can also affect the results. Health professionals could increase bias in their answers because they may have already experienced FA training and may be more motivated and more experienced than a layperson. If the sample were composed only of laypersons, it could have largely affected the results. For a more comprehensive picture, experimental research in this field would have to be conducted. Based on the findings, it would be reasonable to create and evaluate an adapted FA training course for older adults.

## Supplemental Material

sj-docx-1-heb-10.1177_10901981211026531 – Supplemental material for Tailoring First Aid Courses to Older Adults Participants Click here for additional data file. Supplemental material, sj-docx-1-heb-10.1177_10901981211026531 for Tailoring First Aid Courses to Older Adults Participants by Eva Dolenc, Marko Kolšek, Damjan Slabe and Ivan Eržen in Health Education & Behavior

sj-docx-2-heb-10.1177_10901981211026531 – Supplemental material for Tailoring First Aid Courses to Older Adults Participants Click here for additional data file. Supplemental material, sj-docx-2-heb-10.1177_10901981211026531 for Tailoring First Aid Courses to Older Adults Participants by Eva Dolenc, Marko Kolšek, Damjan Slabe and Ivan Eržen in Health Education & Behavior

sj-docx-3-heb-10.1177_10901981211026531 – Supplemental material for Tailoring First Aid Courses to Older Adults Participants Click here for additional data file. Supplemental material, sj-docx-3-heb-10.1177_10901981211026531 for Tailoring First Aid Courses to Older Adults Participants by Eva Dolenc, Marko Kolšek, Damjan Slabe and Ivan Eržen in Health Education & Behavior
