# Supplementary material for: Tailoring First Aid Courses to Older Adults Participants
Source: Health Educ Behav. 2021 Aug 5;49(4):697–707. doi: 10.1177/10901981211026531 (PMC9350451; doi:10.1177/10901981211026531)
Supplement: sj-docx-1-heb-10.1177_10901981211026531 – Supplemental material for Tailoring First Aid Courses to Older Adults Participants [file sj-docx-1-heb-10.1177_10901981211026531.docx]

**Appendix A**

**QUESTIONNAIRE: OLDER ADULTS AND FIRST AID**

**Introductory questions**

1. What do you imagine if I say first aid? What comes to your mind?
2. Please, tell me briefly your opinion about first aid?

**Questions about experiences**

1. Have you ever visited a first aid course? Can you tell me what do you remember about the course?
2. Optional: How do you deal with or have you dealt with first aid? What is your experience in this field?
3. Optional: Do you have any experiences in the field of first aid with an emphasis on older adults? Which ones?
4. Where did people, when you were still a child or a teenager, get knowledge about health, illness, and first aid? (Sub-questions: Where did you find out what to do if you get burned? Did anyone tell you, or you read about it somewhere?)
5. Have you ever been injured or seriously ill? Can you tell me more about how it was? (Sub-questions: Did this in any way affect you, did you learn anything from it, was there anything positive in it?)
6. Do you have any experience, or were you there when someone was injured in an accident or got seriously ill? Please describe this experience to me briefly. What else do you remember?

**Questions about first aid training**

1. Please present to me briefly what do you think about the current situation of first aid training for older adults in Slovenia. (Sub-questions: Do we take care of them? Are they motivated to acquire knowledge?)
2. If an accident in which someone is injured or sick occurs, do you think that even people over the 60' should be taught how to deal with the injured or sick person before the arrival of doctors or paramedics?
3. According to the previous question: What about people over 80 or 90? Should we train them also? If yes, what?
4. What would you like to learn about health, injuries, and accidents? In what would you be most interested?
5. Do you believe that older people have enough strength and concentration to attend a course where they would learn about first aid for approximately two hours? (With sub-questions: 70 years old ones, what about people over 80 or 90?)
6. Do you have any ideas, suggestions on learning first aid for older adults? (With sub-questions: What would you teach them, how, of what age, by whom?)
7. How do you take care of your health nowadays in order not to get injured or sick?
