# Supplementary material for: Tailoring First Aid Courses to Older Adults Participants
Source: Health Educ Behav. 2021 Aug 5;49(4):697–707. doi: 10.1177/10901981211026531 (PMC9350451; doi:10.1177/10901981211026531)
Supplement: sj-docx-2-heb-10.1177_10901981211026531 – Supplemental material for Tailoring First Aid Courses to Older Adults Participants [file sj-docx-2-heb-10.1177_10901981211026531.docx]

**Appendix B**

**A CHECKLIST: Crucial items which are good to consider when planning first aid course for older adults.**
